# Incidence and predictors of stroke and silent cerebral embolism following very high-power short-duration atrial fibrillation ablation

**DOI:** 10.1093/europace/euad327

**Published:** 2023-11-01

**Authors:** Márton Boga, Ferenc Imre Suhai, Gábor Orbán, Zoltán Salló, Klaudia Vivien Nagy, Levente Szegedi, Zsófia Jokkel, Judit Csőre, István Osztheimer, Péter Perge, Dhiraj Gupta, Béla Merkely, László Gellér, Nándor Szegedi

**Affiliations:** Heart and Vascular Center, Semmelweis University, Városmajor u. 68., Budapest 1122, Hungary; Heart and Vascular Center, Semmelweis University, Városmajor u. 68., Budapest 1122, Hungary; Heart and Vascular Center, Semmelweis University, Városmajor u. 68., Budapest 1122, Hungary; Heart and Vascular Center, Semmelweis University, Városmajor u. 68., Budapest 1122, Hungary; Heart and Vascular Center, Semmelweis University, Városmajor u. 68., Budapest 1122, Hungary; Heart and Vascular Center, Semmelweis University, Városmajor u. 68., Budapest 1122, Hungary; Heart and Vascular Center, Semmelweis University, Városmajor u. 68., Budapest 1122, Hungary; Heart and Vascular Center, Semmelweis University, Városmajor u. 68., Budapest 1122, Hungary; Heart and Vascular Center, Semmelweis University, Városmajor u. 68., Budapest 1122, Hungary; Heart and Vascular Center, Semmelweis University, Városmajor u. 68., Budapest 1122, Hungary; Liverpool Heart and Chest Hospital, University of Liverpool, Liverpool, UK; Heart and Vascular Center, Semmelweis University, Városmajor u. 68., Budapest 1122, Hungary; Heart and Vascular Center, Semmelweis University, Városmajor u. 68., Budapest 1122, Hungary; Heart and Vascular Center, Semmelweis University, Városmajor u. 68., Budapest 1122, Hungary

**Keywords:** Very high-power short-duration, Stroke, Silent cerebral embolism, High-power short-duration, Pulmonary vein isolation, Atrial fibrillation

## Abstract

**Aims:**

Cerebral thrombo-embolism is a dreaded complication of pulmonary vein isolation (PVI) for atrial fibrillation; its surrogate, silent cerebral embolism (SCE) can be detected by diffusion-weighted brain magnetic resonance imaging (bMRI). Initial investigations have raised a concern that very high-power, short-duration (vHPSD; 90 W/4 s) temperature-controlled PVI with the QDOT Micro catheter may be associated with a higher incidence of SCE compared with low-power long-duration ablation. We aimed to assess the incidence of procedural complications of vHPSD PVI with an emphasis on cerebral safety.

**Methods and results:**

We enrolled 328 consecutive patients undergoing their PVI procedure using vHPSD. A subgroup of 61 consecutive patients underwent diffusion-weighted bMRI within 24 h of the procedure, and incidence and predictors of SCE were studied. The mean procedure time and left atrial dwell time for the overall cohort were 69.6 ± 24.1 and 46.5 ± 21.5 min, respectively. First-pass isolation was achieved in 82%. No stroke or transient ischaemic attack occurred. Silent cerebral embolism was identified in 5 of 61 patients (8.2%). Silent cerebral embolism following procedures was significantly associated with lower baseline generator impedance (105.8 vs. 112.6 Ω, *P* < 0.0001) and with intermittent loss of catheter–tissue contact during ablation (14.1% vs. 6.1%, *P* < 0.0001).

**Conclusion:**

Very high-power, short-duration PVI is a safe technique with an excellent acute success rate. Silent cerebral embolism incidence in our cohort was below the previously reported range, with no clinically overt cerebral complications. Lower baseline generator impedance and loss of contact during ablation may contribute to a higher risk of SCEs.

## Graphical Abstract

**Graphical abstract:**
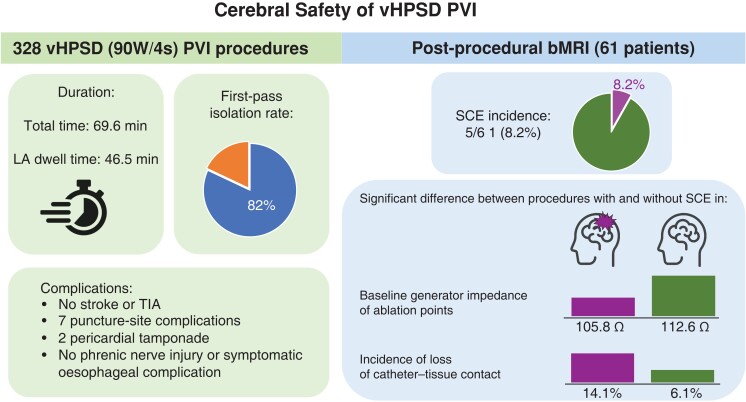

**What’s new?**

- To date, this is the largest study reporting on the safety and acute efficacy of pulmonary vein isolation using the very high-power short-duration (vHPSD) technique.
- The vHPSD ablation technology has an excellent cerebral safety profile with low silent cerebral embolism (SCE) rates and good acute procedural efficacy.
- Lower baseline generator impedance, lower contact force, and intermittent loss of contact during ablation are associated with SCEs.

## Introduction

Atrial fibrillation (AF) is the most common sustained cardiac arrhythmia linked to higher morbidity and mortality rates and decreased quality of life.^1^ Pulmonary vein isolation (PVI) using radiofrequency (RF) is accepted as an effective method of rhythm control therapy for AF. An important recent advancement in RF ablation is very high-power, short-duration (vHPSD) ablation,^2^ using a power output of 90 W in a temperature-controlled mode for a maximum of 4 s, marking a significant advancement from previous low-power long-duration (LPLD; 25–35 W for ∼20–30 s) and high-power short-duration (HPSD; 50 W for ∼10–15 s) ablation techniques. Efficacy and oesophageal safety of PVI with vHPSD have already been demonstrated previously.^3–5^

Clinically overt stroke and transient ischaemic attack (TIA) are severe but rare complications associated with PVI. Meanwhile, silent (asymptomatic) cerebral embolism (SCE) occurs more frequently and is detectable by diffusion-weighted brain magnetic resonance imaging (bMRI), indicating the risk of embolism associated with a given ablation technology.^6,7^ Two initial investigations of vHPSD ablation showed an unexpectedly high incidence of SCE of 24–26%.^5,8^ In comparison, reported SCE rates for LPLD ablation are much lower (6–16%).^9–11^

Given that vHPSD ablation results in shorter left atrial dwell time and smaller lesion size, with a lower incidence of steam pops, the higher SCE incidence compared with LPLD ablation was not anticipated.^12,13^ We hypothesized that vHPSD PVI with short left atrial dwell time and appropriate intra-procedural anticoagulation results in low rates of symptomatic and asymptomatic cerebral lesions. Therefore, we conducted a large-scale prospective evaluation of vHPSD PVI, with a focus on its cerebral safety.

## Methods

In the current single-centre, prospective, observational study, we enrolled 328 patients undergoing their first-time PVI procedure using the QDOT Micro catheter (Biosense Webster, Inc., Irvine, CA, USA) in the QMODE+ setting (90 W/4 s). Inclusion and exclusion criteria are listed in the Supplementary material online. In a subgroup of patients, bMRI was performed within 24 h of the procedure to detect potential SCE. Patients provided written, informed consent to the ablation procedure, post-ablation bMRI, data retrieval, and analysis. Ethics approval was obtained from the Hungarian National Public Health and Medical Officer Service (38843-5/2022/EÜIG).

### Study endpoints

The primary endpoints of the current study were procedure-related cerebral complications, including stroke, TIA, and SCE. Secondary endpoints were the following procedural parameters: procedure time, left atrial dwell time, and first-pass isolation (FPI) rate. In the bMRI subgroup, further parameters were registered, including the number of RF applications, RF time, amount of irrigation fluid, electrical cardioversion (ECV), and activated clotting time (ACT). In the bMRI subgroup, we exported and analysed the following parameters of all RF applications: power, catheter tip temperatures, contact force (CF), and generator impedance. Loss of CF during RF application was defined as a minimum CF of 0 g.

### Pre-ablation protocol

To exclude left atrial appendage (LAA) thrombus, all patients underwent computed tomography angiography (CTA) within 48 h before the procedure. If CTA was unable to rule out LAA thrombus, transoesophageal echocardiography was performed. All patients were on non-vitamin K antagonist oral anticoagulants and one single dose was omitted on the morning of the procedure and administration was resumed 4 h after the procedure.

### Pulmonary vein isolation procedure

**Figure 1:**
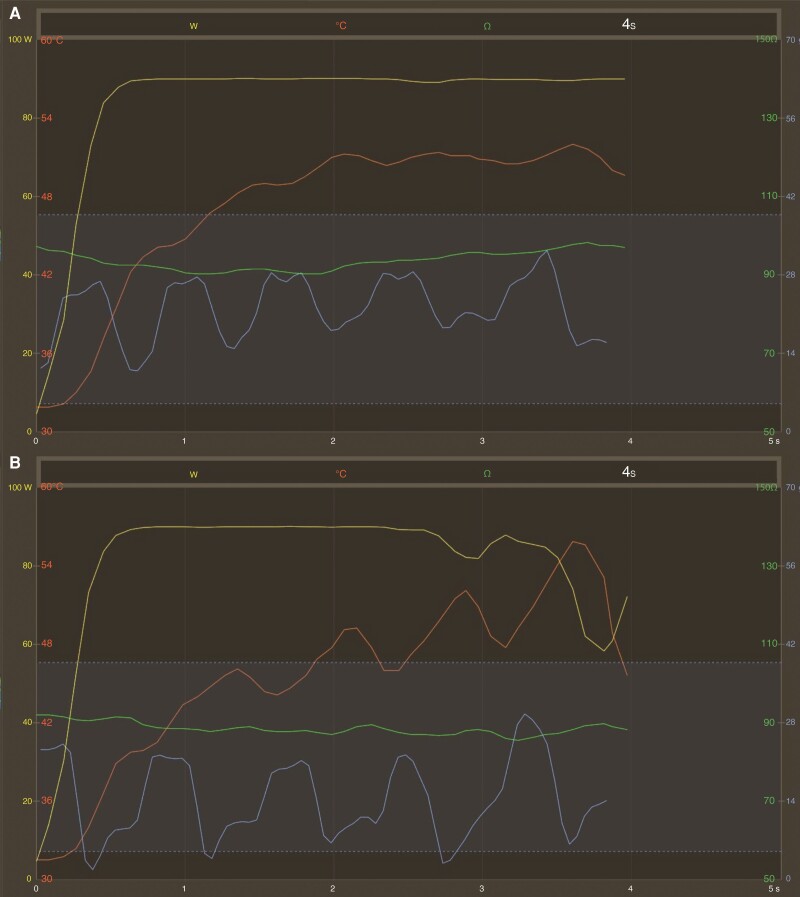
Graph showing the changes of ablation parameters during the 4-s applications with QMODE+ setting (90 W/4 s, temperature-controlled mode). Yellow: power (W); red: temperature (°C); blue: contact force (g); green: impedance (Ω). (*A*) The target temperature of 55°C is not reached, stable 90 W of power is delivered for 4 s. (*B*) The target temperature is reached, power is down-regulated to prevent overheating.

**Figure 2:**
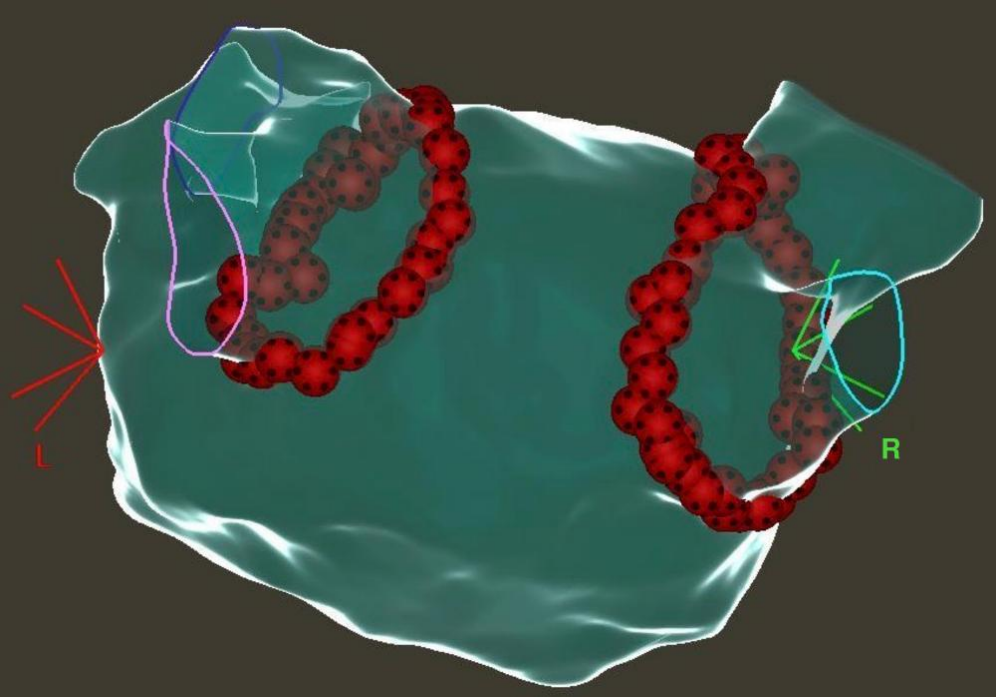
Anatomical map of the left atrium after PVI (posteroanterior view). PVI, pulmonary vein isolation.

The catheter ablation was carried out under conscious sedation with midazolam, propofol, and fentanyl. Procedures were performed by experienced operators, each of whom had performed >300 previous procedures. After femoral venous access, a fluoroscopy and pressure monitoring-guided double trans-septal puncture was performed. Intravenous heparin was administered according to body weight after the first trans-septal puncture and guided by ACT measurements every 20 min; the target value was a minimum of 300 s. A fast anatomical left atrial map was created with an electroanatomical mapping system (CARTO 3, Biosense Webster Inc, Diamond Bar, CA, USA) using a multipolar mapping catheter (either Lasso or PentaRay, Biosense Webster Inc). Point-by-point PVI was performed with a steerable sheath, QDOT Micro catheter (Biosense Webster Inc), and an nGEN generator using the QMODE+ setting for the whole procedure (90 W/4 s, temperature-controlled mode). The neutral electrode patch was positioned at the lower back of the patient. Considering that lesions with vHPSD are smaller, our targeted inter-lesion distance was 5 mm posteriorly and <5 mm anteriorly.^3,12^ The QDOT Micro catheter is a CF sensing RF ablation catheter with six thermocouples embedded in the tip for precise local temperature measurement. The vHPSD algorithm modulates power based on the temperature measured by the thermocouples, with a target temperature of 55°C. If the target temperature is not reached, stable 90 W power is delivered for 4 s (*Figure 1A*); while if it is reached, power is down-regulated to prevent overheating (*Figure 1B*).^14^ After creating the first-pass circle, the presence or absence of FPI was assessed by multipolar catheters. If PVI was incomplete at this point, touch-up applications were delivered until both exit and entrance blocks were reached for all pulmonary veins. A representative anatomical map of a PVI with QMODE+ is shown in *Figure 2*. No additional arrhythmia substrates were targeted beyond PVI. There were no catheter exchanges through the sheaths to prevent air intrusion.

### Post-ablation brain magnetic resonance imaging

A subgroup of consecutive patients who did not have a contraindication for MRI, and whose procedures were performed between July 2022 and April 2023, underwent bMRI within 24 h of the PVI procedure. Magnetic resonance imaging examinations were performed on a 1.5 T MR scanner (Magnetom Aera, Siemens) using a 20-channel head coil. We used the same MRI protocol as described previously.^15^ Diffusion MRI acquisitions were performed using a single shot spin echo, echo-planar imaging sequence in three diffusion encoding directions with b = 1000 s/mm^2^ and one b = 0 measurement repetition time/echo time, 9000 ms/88 ms. Whole brain coverage was obtained with 5 mm thick contiguous axial slices. Diffusion-weighted images (DWIs) and apparent diffusion coefficient (ADC) maps were used to detect SCE.

**Figure 3:**
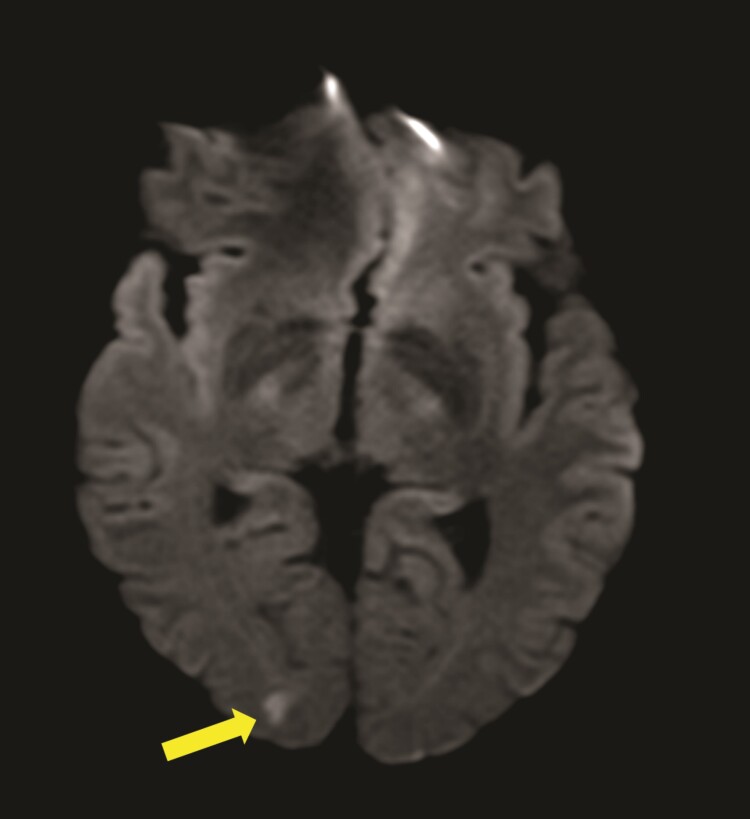
SCE on the diffusion-weighted MR sequence (arrow). MR, magnetic resonance; SCE, silent cerebral embolism.

Silent cerebral embolisms were defined as new ischaemic lesions showing restricted diffusion on the DWI (*Figure 3*) and ADC dataset. The localization, number, and size in three perpendicular diameters of all lesions were recorded using an AGFA PACS workstation (Impax 6.5.2.657, Agfa HealthCare).

### Statistics

Continuous variables are presented as mean and standard deviation or median and inter-quartile range, and their distribution was tested with the Shapiro–Wilk test. Student’s *t*-test and Mann–Whitney *U* test were used for unpaired group comparison. Categorical variables are presented as frequency and percentage and were compared by Fisher’s exact test. Optimal cut-off values were established using receiver operating characteristic (ROC) analysis. Statistical analyses were performed using GraphPad Prism 8 (GraphPad Softwares Inc., USA). A two-tailed *P*-value of <0.05 was considered statistically significant.

## Results

### Study population

The study enrolled 328 consecutive patients who underwent their first PVI procedure. *Table 1* summarizes the baseline characteristics of the study population. For the whole cohort, the mean age was 62 ± 14 years, 36% were female, and 70% had paroxysmal AF. Sixteen (5%) patients had a history of stroke or TIA prior to the procedure. The average CHA_2_DS_2_-VASc score was 3 ± 2.

**Table 1:** Baseline characteristics of the whole study population and the bMRI subgroup of this population separated into patients with and without SCE

	All patients (*N* = 328)	Patients in the bMRI subgroup (*N* = 61)	
		With SCE (*N* = 5)	Without SCE (*N* = 56)
Age, years	62 ± 14	60 ± 11	62 ± 11
Sex, female (%)	118 (36)	2 (40)	14 (25)
BMI, kg/m^2^	28.9 ± 4.4	27.4 ± 4.9	29.4 ± 4.9
CHA_2_DS_2_-VASc score	3 ± 2	2 ± 1	2 ± 1
Paroxysmal AF, *n* (%)	231 (70)	5 (100)	38 (68)
LVEF, %	57 ± 8	61 ± 7	56 ± 6
Hypertension, *n* (%)	222 (68)	2 (40)	33 (59)
Diabetes, *n* (%)	53 (16)	0 (0)	5 (9)
CAD, *n* (%)	61 (19)	0 (0)	8 (14)
Prior stroke/TIA, *n* (%)	16 (5)	0 (0)	3 (5)
CHF, *n* (%)	16 (5)	0 (0)	3 (5)
LA diameter, mm	48 ± 7	51 ± 3	48 ± 6

Continuous variables are expressed as mean ± standard deviation, and categorical variables are expressed as numbers and percentages.

AF, atrial fibrillation; BMI, body mass index; bMRI, brain magnetic resonance imaging; CAD, coronary artery disease; CHF, chronic heart failure; LA, left atrium; LVEF, left ventricular ejection fraction; SCE, silent cerebral embolism; TIA, transient ischaemic attack.

### Procedural data

The mean procedure time and left atrial dwell time for the whole cohort were 69.6 ± 24.1 and 46.5 ± 21.5 min, respectively. Intra-procedural cardioversion was performed in 68 (20%) procedures.

In the bMRI subgroup, the mean number of RF applications was 79 ± 21, with a total RF application time of 309 ± 85 s. The mean volume of irrigation fluid was 146 ± 42 mL, and the average ACT was 324 ± 38 s.

### Efficacy results

For the whole study population, acute PVI success rate was 100%, and FPI was achieved in 82% of the patients. The rate of freedom from arrhythmia at 6 months by standard-of-care monitoring was 84.5%.

### Procedural safety

No stroke or TIA occurred as a complication of the 328 procedures. There were seven puncture site complications, including one arteriovenous fistula, one pseudoaneurysm, and five cases of groin haematoma. Pericardial tamponade occurred in two cases, both resolved with percutaneous pericardiocentesis. No phrenic nerve injury or symptomatic oesophageal complication occurred. There were no audible steam pops during the procedures.

Patients in the SCE screening subgroup underwent bMRI after a median of 18 (16–22) h.

Silent cerebral embolism was detected in 5 of 61 patients, corresponding to an incidence rate of 8.2%. Four patients showed only one DWI-positive lesion ranging from 2 to 9 mm. One patient showed multiple 2–4 mm lesions in the frontal lobe. None of these patients exhibited any clinical neurological signs.

**Figure 4:**
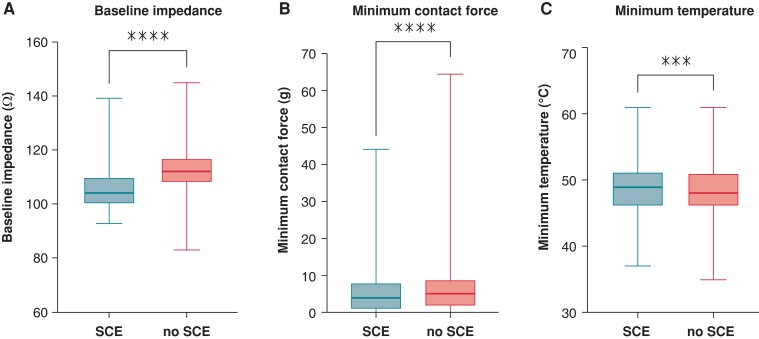
Baseline impedance (*A*), minimum contact force (*B*), and maximum temperature (*C*) of ablation points in procedures with and without SCE. SCE, silent cerebral embolism, ***P < 0.001, ****P < 0.0001.

There was no significant difference between procedures with and without SCE in terms of procedural parameters (*Table 2*). A total of 4773 ablation points were analysed (*Table 3*). In procedures with SCE, baseline generator impedance (*Figure 4A*) and minimum CF (*Figure 4B*) were significantly lower (105.8 vs. 112.6 Ω, *P* < 0.0001 and 5.9 vs. 7.1 g, *P* < 0.0001), and the incidence of loss of catheter–tissue contact was significantly higher (14.1% vs. 6.1%, *P* < 0.0001). Maximum temperature (*Figure 4C*) and temperature rise of ablation points were also significantly higher in procedures with SCE (49.2 vs. 48.4°C, *P* = 0.0010 and 15.2 vs. 14.3°C, *P* < 0.0001, respectively).

**Table 2:** Parameters of procedures with and without SCE

	Procedures with SCE (*N* = 5)	Procedures without SCE (*N* = 56)	*P*-value
Procedure time, min	55.4 ± 15.2	61.2 ± 15.6	0.43
Left atrial dwell time, min	41.2 ± 15	43.6 ± 13	0.69
RF time, s	322 ± 127	307 ± 81	0.72
RF applications, *n*	82 ± 31	79 ± 21	0.73
Irrigation, mL	150 ± 59	145 ± 41	0.80
FPI, %	100	80	>0.99
ECV, %	40	11	0.12
ACT, s	336 ± 32	323 ± 38	0.41

Continuous variables are expressed as mean ± standard deviation, and categorical variables are expressed as numbers and percentages.

ACT, activated clotting time; ECV, electrical cardioversion; FPI, first-pass isolation; RF, radiofrequency; SCE, silent cerebral embolism.

**Table 3:** Parameters of ablation points in procedures with and without SCE

	Ablation points in procedures with SCE (*n* = 410)	Ablation points in procedures without SCE (*n* = 4363)	*P*-value
Mean power, W	81.7 ± 5	82.3 ± 3.8	0.1867
Minimum temperature, °C	33.9 ± 1.6	34.1 ± 1.5	**0.0010**
Maximum temperature, °C	49.2 ± 3.9	48.4 ± 3.9	**0**.**0009**
Temperature rise, °C	15.2 ± 3.9	14.3 ± 4	**<0.0001**
Baseline impedance, Ω	105.8 ± 8.3	112.6 ± 7.5	**<0.0001**
Impedance drop, Ω	8.5 ± 3.5	8.6 ± 3.3	0.1088
Minimum CF, g	5.9 ± 6.3	7.1 ± 6.8	**<0.0001**
Maximum CF, g	28 ± 15.7	27.8 ± 16.7	0.4135
Mean CF, g	15.1 ± 9	15.6 ± 9.5	0.2346
Loss of contact (CF minimum = 0), *n* (%)	58 (14.1)	273 (6.1)	**<0.0001**

Continuous variables are expressed as mean ± standard deviation, and categorical variables are expressed as numbers and percentages.

CF, contact force; SCE, silent cerebral embolism; bold values indicate *p* < 0.05.

**Figure 5:**
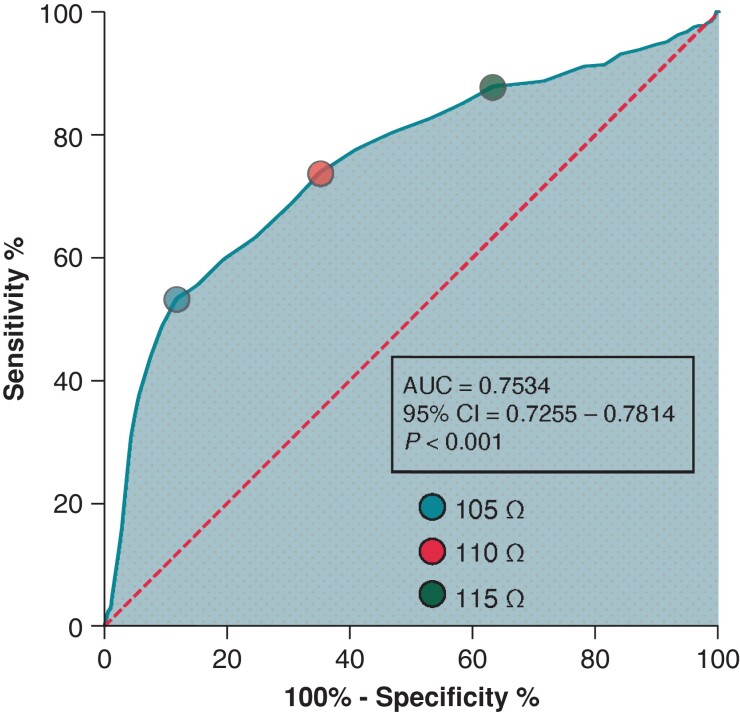
ROC curve of baseline generator impedance for predicting SCE. AUC, area under curve; CI, confidence interval; ROC, receiver operator characteristic; SCE, silent cerebral embolism.

To determine the optimal cut-off value for baseline generator impedance that marks a safety threshold for SCE risk, we performed ROC analysis [area under curve (AUC) = 0.7534, 95% CI = 0.7255–0.7814, *P* < 0.0001; *Figure 5*]. A baseline impedance of 110 Ω appeared to be an optimal cut-point (sensitivity: 73.9%, specificity: 64.9%, positive predictive value: 16.5%, negative predictive value: 96.4%).

The probability of SCE was higher in case of ablation points with loss of contact [odds ratio (OR) = 2.51; *P* < 0.0001] and baseline impedance <110 Ω (OR = 5.23; *P* < 0.0001).

## Discussion

### Main message

To the best of our knowledge, this is the largest study reporting on the acute efficacy and safety of PVI using the vHPSD technique. In addition to short procedure and RF delivery times, we found an 82% rate of FPI, and no clinically manifest cerebral complications. Furthermore, compared with previous studies,^5,8^ a low SCE rate of 8.2% was observed. Lower baseline generator impedance, lower contact force, and intermittent loss of contact during ablation were associated with SCEs, thereby offering a potential way to prevent cerebral complications in the future.

### Very high-power, short-duration ablation

The introduction of vHPSD is potentially a significant advancement in RF ablation technology, offering the potential for impressively fast procedures due to short, 4-s application times per point. Indeed, previous studies have shown that ablation with vHPSD results in significantly shorter procedure times than LPLD and HPSD ablation.^3,4,16^ High-energy ablation is intended to induce more resistive and minimal conductive heating than lower power ablation, leading to more consistent lesion formation. However, for vHPSD to gain wider traction, these benefits need to be accompanied by at least equivalent efficacy and safety outcomes as described for low-power RF ablation.

One of the aims of vHPSD technology is to improve the safety profile of RF ablation by creating a very well-regulated ablation environment with rapid temperature feedback, power regulation, and continuous irrigation. Tissue temperatures with 90 W are reported to be lower, and lesions were shown to be shallower but still deep enough to provide transmurality in the atria.^12^ Furthermore, it is the potentially shorter left atrial dwell time associated with vHPSD ablation that may contribute to higher safety by reducing embolic events.^17^

### Clinical significance of silent cerebral embolism

Silent cerebral embolisms detected by diffusion-weighted bMRI can serve as a surrogate marker for symptomatic cerebrovascular events, enabling comparison of the thrombogenicity of various AF ablation techniques.^6,7^ Evidence suggests that SCEs may have significant long-term implications for neurocognitive function, manifesting in an increased risk for dementia.^18^ Recent data showed that even over a shorter timeframe, silent brain infarcts could have a similar impact on cognitive decline as symptomatic cerebral events.^19^

### Post-ablation brain magnetic resonance imaging studies

Several studies have examined the rate of SCE after PVI using LPLD (25–35 W) ablation, showing an incidence of 6–16%.^9–11^ Pre-procedural baseline bMRI was performed in several investigations; however, the incidence rate of novel SCE did not differ significantly from the findings of research without baseline bMRI, indicating that post-procedural DWI bMRI in itself is an adequate method to detect ablation-related SCEs.

The first-ever study of the vHPSD approach was the QDOT-FAST Trial, in which SCEs were identified in 6 of 51 (12%) patients who underwent post-procedural bMRI.^2^ All except one lesion were resolved at a 1-month follow-up bMRI. An investigation by Halbfass *et al.*^5^ evaluated the safety of vHPSD ablation with regards to silent oesophageal injury and SCE. The reported mean procedure time was 96 min, and the FPI rate was only 43%. A subgroup of 21 patients, whose procedure was performed using the nGEN generator, underwent bMRI. Charring at the catheter tip was observed in 11%, and SCEs were present in 5 of 21 patients (24%). Towards the end of 2020, a safety notice was published by Biosense Webster after a high number of catheter char reports using QMODE+ with the nGEN generator. Subsequently, a software update was released that included modifications to optimize the temperature-controlled algorithm. The same German research group reassessed the safety aspects using the modified RF generator.^8^ In this investigation, the SCE rate was still high (6 of 23 patients, 26%). These two studies had limited sample sizes and did not report the utilized peri-procedural anticoagulation protocol nor the ACT values during procedures. Ablation at the posterior wall was performed with only 3-s applications in both studies, and the target inter-lesion distance was <6 mm, which might be the reason for the low FPI rate. The most recent investigation into the incidence of SCE following vHPSD ablation was conducted by Kottmaier *et al.*,^20^ describing a 17% SCE rate in 23 patients.

In the current study, we identified five patients with SCE but did not observe any clinically overt cerebrovascular events. The results suggest that vHPSD PVI is a safe procedure in terms of cerebral complications, with a safety profile comparable with that of LPLD ablation. The SCE rate of our current finding is significantly lower than previously reported with vHSPD. Possible explanations for this difference are the following: (i) longer left atrial dwell times reported in previous papers; (ii) ACT levels (not reported previously); (iii) catheter instability and lack of CF during RF delivery; and (iv) baseline generator impedance (Mueller *et al.*^8^ reported that the formation of catheter-tip coagulum ceased when repositioning the neutral electrode so that impedance changed from 90 to 110 Ω).^5^ There were no notable differences between the methodologies of these studies, and similarly to ours, all of the investigations were carried out using a 1.5 T MRI device.

### Predictors of silent cerebral embolism

Procedural parameters (long procedure time, intra-procedural ECV, low ACT level, and the type of electroanatomical mapping system) are known factors that influence SCE rate.^17,21^ Interrupted anticoagulation has also been reported to increase the incidence of SCE.^22,23^ It should be noted that the literature lacks agreement on whether SCEs result from solid or gaseous emboli. A clear ‘either-or’ explanation is unlikely due to the large spectrum of potential causes, e.g. coagulum formation at ablation sites or on the catheter, entry of air through sheaths, and gas bubble formation during ablation.^24^ Nevertheless, most of the predictors mentioned above support the thrombo-embolic hypothesis.

Our results demonstrate that lower baseline generator impedance and loss of contact during ablation are also associated with the incidence of SCE. Bourier *et al.*^25^ showed that generator impedance plays a crucial role in determining current delivery and varies significantly among patients—they observed lower impedance in males and patients with lower BMI. Radiofrequency lesions are created through the delivery of current, not power. Consequently, impedance should be a factor taken into account as it significantly influences the effect of ablation, with lower impedance resulting in higher current flow. The latter may result in higher tissue temperatures, which can lead to a higher risk of coagulum (or bubble) formation. This aligns with the observation of Mueller *et al.*^8^ that catheter-tip coagulum formation completely ceased when increasing baseline impedance to 110 Ω. Similarly, we also found that a mean baseline impedance of 110 Ω during the procedure is an optimal target. Noteworthy, we found that the measured baseline impedance before the first application consistently tends to be slightly higher (with a mean difference of 5 Ω), possibly due to the effect of ablation on the impedance at neighbouring points. Therefore, it seems reasonable to position the neutral electrode to reach a target baseline impedance of >115 Ω before the first RF application in patients with lower initial generator impedance to reduce the risk of SCE.

Catheter–tissue contact is a crucially important factor in the case of vHPSD ablation, as all of the current is delivered within a really short 4-s timeframe, wherein even a momentary loss of contact may lead to a substantial reduction in current transmission to the myocardial tissue. Apart from affecting the efficacy of ablation, this may also contribute to a higher risk of SCEs, based on our results. Plausibly, this observation might be attributed to the effect of the higher local current delivered to the blood pool in the left atrium resulting in coagulum (or bubble) formation and charring. This could potentially elucidate the low rates of FPI and high rates of SCE in the earlier studies about vHPSD.^5,8^

### Limitations

The present study has some limitations that need to be acknowledged. It is a single-centre study with six operating physicians. The selection of patients for bMRI was not randomized. Consecutive patients were enrolled who did not have contraindication for MRI examination and gave written consent. The sample size in the bMRI subgroup was moderate, but still, this is the largest study about vHPSD, also investigating cerebral complications of vHPSD PVI. The study protocol did not include a pre-intervention baseline MRI, as the DWI sequence is able to detect acute lesions, and thus differentiation from chronic lesions does not pose an issue. Lastly, no neurological follow-up was carried out to evaluate cognitive function outcomes.

## Conclusion

Our results verify that vHPSD RF ablation technology has a good cerebral safety profile with low SCE rates and good acute procedural efficacy. Lower baseline generator impedance and loss of contact during ablation may contribute to a higher risk of SCEs.

## Supplementary material

Supplementary material is available at *Europace* online.

## Supplementary Material

euad327_Supplementary_Data

## Data Availability

The data underlying this article cannot be shared publicly due to privacy/ethical reasons (General Data Protection Regulation). The data will be shared on reasonable request to the corresponding author.
