# Supplementary material for: Incidence and predictors of stroke and silent cerebral embolism following very high-power short-duration atrial fibrillation ablation
Source: Europace. 2023 Nov 1;25(11):euad327. doi: 10.1093/europace/euad327 (PMC10653180; doi:10.1093/europace/euad327)
Supplement: euad327_Supplementary_Data [file euad327_supplementary_data.docx]

**Supplemental material for publication: Incidence and Predictors of Stroke and Silent Cerebral Embolism Following Very High-Power Short-Duration Atrial Fibrillation Ablation**

Márton Boga*^1^, MD; Ferenc Imre Suhai*^1^, MD, PhD; Gábor Orbán^1^, MD; Zoltán Salló^1^, MD; Klaudia Vivien Nagy^1^, MD, PhD; Levente Szegedi^1^, Zsófia Jokkel^1^, MD; Judit Csőre^1^, MD; István Osztheimer^1^, MD, PhD; Péter Perge^1^, MD, PhD; Dhiraj Gupta^2^, MD, PhD, DSc; Béla Merkely^1^, MD, PhD, DSc; László Gellér^1^, MD, PhD, DSc; Nándor Szegedi^1^, MD, PhD

*these two authors contributed equally to the paper

^1^Heart and Vascular Center, Semmelweis University

^2^Liverpool Heart and Chest Hospital, University of Liverpool

| Patient inclusion criteria | Symptomatic paroxysmal / persistent / long-standing persistent atrial fibrillation |
| --- | --- |
|  | Age over 18 years |
|  | Voluntary consent to participate |
| Patient exclusion criteria | Contraindication for anticoagulation |
|  | Contraindication for ablation |
|  | Contraindication for MRI |
|  | Poor image quality |
|  | Previous PVI or heart surgery |
|  | Pregnancy |
|  | Life expectancy < 1 year |
|  | Valvular AF |
|  | Reversible cause of AF |
|  | Further ablation beyond PVI |

Patient inclusion and exclusion criteria
